# Novel diagnostic biomarkers for pancreatic cancer: assessing methylation status with epigenetic-specific peptide nucleic acid and KRAS mutation in cell-free DNA

**DOI:** 10.3389/fonc.2024.1395473

**Published:** 2024-07-05

**Authors:** Hongsik Kim, Jinah Chu, In-Gu Do, Yong-Pyo Lee, Hee Kyung Kim, Yaewon Yang, Jihyun Kwon, Ki Hyeong Lee, Chinbayar Batochir, Eunji Jo, Kyo Rim Kim, Hye Sook Han

**Affiliations:** ^1^ Department of Internal Medicine, Chungbuk National University Hospital, Cheongju, Republic of Korea; ^2^ Department of Pathology, Kangbuk Samsung Hospital, Sungkyunkwan University School of Medicine, Seoul, Republic of Korea; ^3^ Department of Internal Medicine, Chungbuk National University Hospital, Chungbuk National University College of Medicine, Cheongju, Republic of Korea; ^4^ Seasun Biomaterials, Daejeon, Republic of Korea

**Keywords:** pancreatic ductal carcinoma, DNA methylation, peptide nucleic acid, biomarker, cell-free DNA

## Abstract

**Purpose:**

Pancreatic ductal adenocarcinoma (PDAC) is an aggressive tumor with a poor prognosis that poses challenges for diagnosis using traditional tissue-based techniques. DNA methylation alterations have emerged as potential and promising biomarkers for PDAC. In this study, we aimed to assess the diagnostic potential of a novel DNA methylation assay based on epigenetic-specific peptide nucleic acid (Epi-sPNA) in both tissue and plasma samples for detecting PDAC.

**Materials and methods:**

The study involved 46 patients with PDAC who underwent surgical resection. Epi-TOP pancreatic assay was used to detect PDAC-specific epigenetic biomarkers. The Epi-sPNA allowed accurate and rapid methylation analysis without bisulfite sample processing. Genomic DNA extracted from paired normal pancreatic and PDAC tissues was used to assess the diagnostic efficacy of epigenetic biomarkers for PDAC. Subsequent validation was conducted on cell-free DNA (cfDNA) extracted from plasma samples, with 10 individuals represented in each group: PDAC, benign pancreatic cystic neoplasm, and healthy control.

**Results:**

The combination of seven epigenetic biomarkers (*HOXA9, TWIST, WT1, RPRM, BMP3, NPTX2*, and *BNC1*) achieved 93.5% sensitivity and 96.7% specificity in discerning normal pancreatic from PDAC tissues. Plasma cfDNA, analyzed using these markers and *KRAS* mutations, exhibited a substantial 90.0% sensitivity, 95.0% specificity, and an overall 93.3% accuracy for discriminating PDAC. Notably, cancer antigen 19-9 and carcinoembryonic antigen both had an accuracy of 90.0%.

**Conclusion:**

Our study suggests that analyzing seven differentially methylated genes with *KRAS* mutations in cfDNA using the novel Epi-TOP pancreatic assay is a potential blood-based biomarker for the diagnosis of PDAC.

## 1 Introduction

Pancreatic ductal adenocarcinoma (PDAC), a highly aggressive tumor, ranks among the leading causes of cancer-related mortality, with a low 5-year survival rate (1, 2). The majority of patients receive a diagnosis at an advanced stage due to the asymptomatic nature or vague symptoms of early-stage PDAC. Although 15% to 20% of them present with resectable PDAC, surgical resection, combined with adjuvant chemotherapy, is the only potentially curative treatment option for early-stage PDAC (3). Therefore, there is an urgent need for highly sensitive, specific, and effective early detection methods to improve the clinical outcomes of pancreatic cancer, particularly using non-invasive samples.

PDAC is a disease that arises from mutations in oncogenes and tumor suppressor genes. Notably, *KRAS* mutations, prevalent in approximately 90% of PDAC cases, play an important role in pancreatic intraepithelial neoplasia and the development of invasive PDAC (4). In addition to genetic mutations, epigenetic alterations such as histone modifications, chromatin accessibility, and DNA methylation also play a critical role in the progression and metastasis of PDAC (5, 6). Therefore, both genetic mutations and epigenetic alterations have been explored as promising biomarkers with potential applications in early detection, monitoring treatment response, selecting treatment targets, and predicting prognosis.

Diagnosing PDAC using traditional tissue-based methods and genetic tests, such as next-generation sequencing, is challenging. In contrast, liquid biopsy offers distinct advantages such as simplicity in sampling, minimal invasiveness, and the ability to capture tumor heterogeneity. Recent advancements in DNA/RNA sequencing and amplification techniques significantly enhance the capability of liquid biopsy for gene sequencing and detecting epigenetic alterations in circulating cell-free DNA (cfDNA) from blood, providing both high sensitivity and specificity (7).

Early diagnosis is of paramount importance in managing early-stage PDAC. Epigenetic alterations play a pivotal role in disease progression and metastasis. Therefore, several prior studies have delved into exploring the potential of diagnostic biomarkers by examining DNA methylation alterations for the early detection of PDAC (8–11). There has been a recent increase in the number of studies focusing on cfDNA methylation analysis as a promising non-invasive approach for the discovery and validation of epigenetic biomarkers with diagnostic or prognostic potential in PDAC (12). Prior to this, several DNA methylation analyses in PDAC have evaluated both at the genome-wide DNA levels and by selected individual genes or small selected individual gene panels (8, 13–20). Despite considerable efforts, a dedicated DNA methylation assay for PDAC diagnosis remains elusive. Furthermore, existing studies have predominantly concentrated on DNA methylation in surgically resected tumor tissue.

Therefore, we aimed to evaluate the diagnostic efficacy of a novel DNA methylation assay using epigenetic-specific peptide nucleic acid (Epi-sPNA) technology in surgical specimens for PDAC and to develop a noninvasive test for detecting DNA methylation in circulating cfDNA from plasma.

## 2 Materials and methods

### 2.1 DNA extraction

Genomic DNA (gDNA) was extracted from paired normal and tumor tissues of 46 patients with PDAC who had undergone surgical resection, using a DNeasy blood and tissue kit (Qiagen, Germany) following the manufacturer’s instructions. Circulating cfDNA was extracted from 2 mL of plasma samples collected from 10 individuals each in the PDAC, benign cystic pancreatic neoplasm, and healthy control groups using a QIAamp Circulating Nucleic Acid Kit (Qiagen, Germany). The extracted gDNA was then stored at -20°C until further analysis. The Institutional Review Board (IRB No. 2023-06-010) at Chungbuk National University Hospital approved this study. Informed consent was obtained from all patients and healthy donors.

### 2.2 Marker screening

Differentially methylated genes in normal pancreatic and PDAC tissues were screened using the Epi-TOP Tumor Suppressor Assay (Seasun Biomaterials, Seoul, Korea) to evaluate the methylation levels of approximately one hundred tumor suppressor genes with differential methylation patterns in solid tumors. Epigenetic biomarkers displaying significant differences in methylation patterns were selected, validated by multiplex analysis, and subsequently used for the detection of PDAC using plasma cfDNA.

### 2.3 Methylation analysis

The methylation status of circulating cfDNA was examined using the Epi-TOP Pancreatic assay (Seasun Biomaterials, Korea), a bisulfite-free real-time polymerase chain reaction (RT-PCR) methylation detection kit targeting selected epigenetic biomarkers that were confirmed to be differentially methylated among normal pancreatic and PDAC tissues. The methylation analysis was conducted following the manufacturer’s instructions. Briefly, after PCR master mix preparation, the PCR reactions were carried out on a CFX96 RT- PCR system (BioRad, USA). Data interpretation involved comparing the cycle threshold (Ct) values among an internal control, target genes in clinical specimens, and an external negative control containing hypomethylated standard DNA following the formula: Percent Methylation Ratio (PMR) = 100/(1 + 2^Ct target − Ct control^). Target genes with higher methylation (PMR) levels than the negative control are classified as hypermethylated.

### 2.4 *KRAS* mutation

Somatic mutations in *KRAS* codon 12, 13 and 61 in plasma specimens were assessed using the U-TOP KRAS detection kit (Seasun Biomaterials, Korea), an RT- PCR assay following the manufacturer’s instructions. Mutations were characterized based on the fluorescence signal of the detection probes and their corresponding Ct values, in comparison with the Ct values of a negative control DNA containing 0.1% mutated *KRAS*.

### 2.5 Data analysis and statistical analysis

The methylation status of the selected genes was examined in PDAC and matched normal pancreatic tissues. Comparisons were made using McNemar’s and chi-square tests. Receiver operating characteristic (ROC) curve analysis and area under the curve (AUC) were used to evaluate the diagnostic efficacy of individual epigenetic markers and combinations of selected genes. The methylation cut-off value for each marker was established based on the highest likelihood ratio. Risk scores were assigned to all patients based on a linear combination of the expression levels of selected genes, carcinoembryonic antigen (CEA), and cancer antigen 19-9 (CA19-9). The weights were determined according to the regression coefficient. Stepwise Cox regression and stratification analyses were performed. A target gene was classified as hypermethylated if the methylation value exceeded a predetermined cutoff value derived from the negative control. A tissue gDNA sample was considered positive for PDAC if at least two of the selected target genes exhibited hypermethylation. For cfDNA, the criteria for distinguishing PDAC included the presence of at least 3 positive markers out of 7 epigenetic biomarkers combined with the presence of a KRAS mutation at either codons 12/13 or 61. Accuracy was determined according to the described in previous publications (21, 22). All P-values were two-sided, and statistical significance was set at P < 0.05. Statistical analyses were performed using R Statistical Software (version 4.0.5; R Foundation for Statistical Computing, Vienna, Austria).

## 3 Results

### 3.1 Patient characteristics and study design

**Figure 1:**
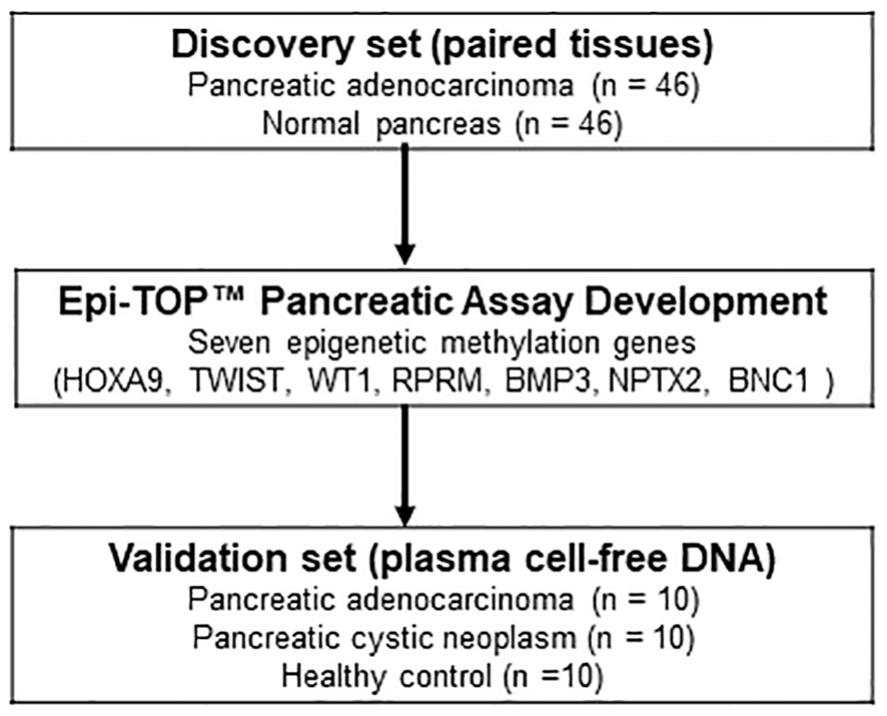
Flow diagram illustrating the study design, delineating the marker discovery set involving tissues and the subsequent validation set utilizing plasma cell-free DNA.

In the discovery set, the qualified tissues from 92 cases (paired 46 PDAC and 46 normal pancreatic tissues, stage I-III) were tested using the assay to evaluate the diagnostic efficacy of epigenetic biomarkers for PDAC. Further validation was performed on cfDNA isolated from 30 plasma samples (10 treatment naïve metastatic PDAC stage IV, 10 benign pancreatic cystic neoplasm without malignant potential, and 10 healthy controls) to evaluate the diagnostic performance of the novel Epi-TOP Pancreatic assay (**Figure 1**). First, we identified novel PDAC-specific epigenetic methylation biomarkers by analyzing the genomic DNA profiles of tissues using the Epi-sPNA method without sample processing using bisulfate. Second, selected epigenetic methylation biomarkers, along with *KRAS* somatic mutations in codons 12/13 and 61 were tested and validated using plasma cfDNA for screening and diagnosis of PDAC. Baseline characteristics of patients at tissue and plasma acquisition are summarized in **Table 1**.

**Table 1:** Baseline patient and sample characteristics.

Pathology	Discovery set (Tissue)	Validation set (Plasma cfDNA)		
	Paired PDAC and normal pancreas	PDAC	Benign pancreatic cystic neoplasm	Healthy
No. of patients	46	10	10	10
Age, median (range),years	67	67 (53-81)	50 (21-80)	47 (40-52)
Gender				
Male	24	5	4	3
Female	22	5	6	7
Clinical stage				
I	3	–	–	–
II	34	–	–	–
III	9	–	–	–
IV	–	10	–	–
KRAS				
Wild type	–	0	10	10
Mutation	–	10	0	0

PDAC, pancreatic ductal adenocarcinoma; cfDNA, cell-free DNA.

"-" not applicable.

### 3.2 Epigenetic methylation biomarkers in the discovery tissue set

**Figure 2:**
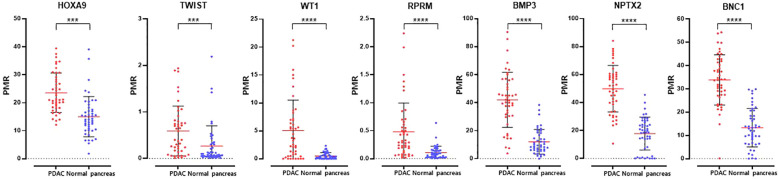
Methylation levels of seven epigenetic DNA methylation markers in tissues of paired PDAC and normal pancreas. ***0.0001 < p ≤ 0.001; ****p ≤ 0.0001.

**Figure 3:**
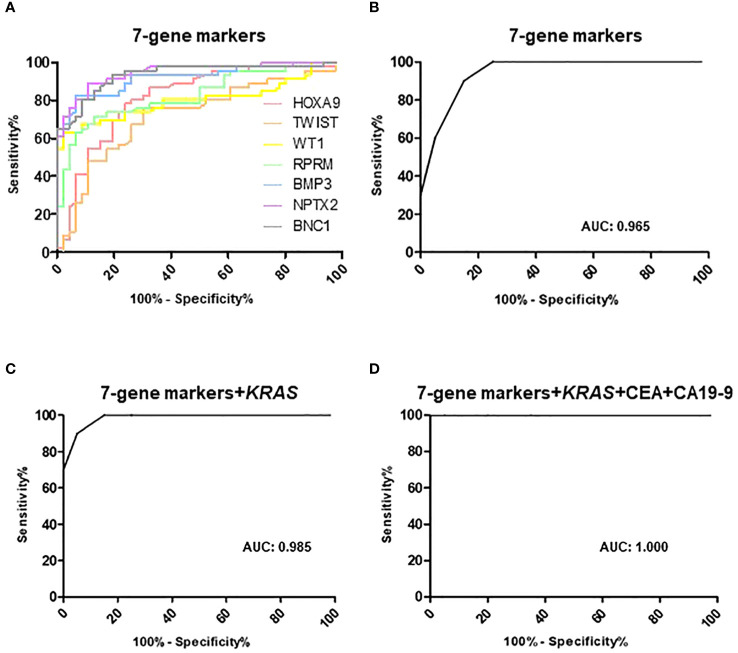
Receiver operator classification curves of **(A)** 7-gene markers, **(B)** combination of 7-gene markers in the discovery tissue set. Receiver operating characteristic curves of **(C)** 7-gene panels with *KRAS* mutation, **(D)** 7-gene panels, *KRAS* mutation, CEA, and CA19-9 in the validation plasma set. Areas under the curves (AUC) are also shown.

We identified differential hypermethylation changes comparing PDAC and normal tissues. We identified seven PDAC-specific DNA gene hypermethylation biomarkers, namely *HOXA9*, *TWIST*, *WT1*, *RPRM*, *BMP3*, *NPTX2*, and *BNC1* (**Figure 2**; **Supplementary Table S1**). The seven hypermethylation biomarkers in the discovery set exhibited AUC values ranging from 0.721 to 0.946 (**Figure 3A**). We constructed a novel 7-gene panel for the Epi-TOP pancreatic assay, with a combination of identified epigenetic biomarkers. The combination of the 7-gene panels (*HOXA9*, *TWIST*, *WT1*, *RPRM*, *BMP3*, *NPTX2*, and *BNC1*) achieved an AUC value of 0.965, with a sensitivity of 93.5% and specificity of 95.7% for distinguishing PDAC (**Table 2**, **Figure 3B**; **Supplementary Table S2**). No significant differences in methylation levels were observed with respect to patient age, sex, or cancer stage (**Supplementary Table S3**).

**Table 2:** Diagnostic performance of Epi-TOP Pancreatic assay with *KRAS* mutation, CEA, CA19-9, and the combined model.

		Cut-off	AUC	95% CI	Sensitivity (%)	Specificity (%)	Accuracy (%)
Discovery set (tissue)	*HOXA9*	25	0.814	0.724-0.904	34.78	93.48	91.72
	*TWIST*	0.5	0.721	0.614-0.827	47.83	86.69	85.78
	*WT1*	2	0.802	0.706-0.898	63.04	97.83	96.78
	*RPRM*	0.5	0.829	0.744-0.913	32.61	97.83	95.87
	*BMP3*	30	0.919	0.862-0.977	78.26	93.48	93.02
	*NPTX2*	40	0.946	0.903-0.990	71.74	97.83	97.04
	*BNC1*	30	0.935	0.883-0.986	65.22	100.00	98.96
	7-gene panel	2/7*	0.965	0.923-1.006	93.48	95.65	94.57
Validation set (plasma cfDNA)	7-gene panel	2/7*	0.943	0.865-1.020	90.00	85.00	86.67
	7 gene panel with *KRAS codon 12/13 or 61*	3/9*	0.985	0.953-1.017	90.00	95.00	93.33
Validation set (serum)	CEA	4	0.915	0.780-1.050	80.00	95.00	90.00
	CA19-9	37	0.970	0.919-1.021	90.00	90.00	90.00
	CEA + CA19-9	1/2*	0.96	0.889-1.031	100.00	90.00	93.33
Validation set (plasma cfDNA + serum)	7 gene panel with *KRAS* *+* CEA + CA19-9	1/3**	0.925	0.826-1.023	100.00	85.00	90.00
		2/3**	1.000	1.000-1.000	100.00	95.00	96.67
	*KRAS*+CEA+CA19-9	1/3**	0.95	0.869-1.031	100.00	90.00	93.33
		2/3**	0.985	0.953-1.017	90.00	95.00	93.33

A/B*, at least ‘A’ out of ‘B’ markers are positive is considered PDAC. A/B**, at least 1 or 2 out of 3 methods (7 gene panel + KRAS, CEA and CA19-9) are positive is considered PDAC.

AUC, Areas under the curves; CI, confidence interval; cfDNA, cell-free DNA; CA19-9, Carbohydrate antigen 19-9; CEA, Carcinoma embryonic antigen.

### 3.3 Diagnostic accuracy of epigenetic methylation biomarkers combined with KRAS mutations in the validation plasma set

To verify the diagnostic accuracy of the Epi-TOP Pancreatic assay in plasma, we analyzed a 7-gene panel along with *KRAS* mutations in circulating cfDNA samples from participants with PDAC, benign pancreatic cystic neoplasm, and healthy controls. The AUC value of the combination 7-gene epigenetic methylation panels and *KRAS* mutation validation test was 0.985 with a high sensitivity of 90.0%, and specificity of 95.0% for discriminating PDAC (**Table 2**, **Figure 3C**; **Supplementary Table S4**).

### 3.4 Comparison of Epi-TOP pancreatic assay with serum CEA and CA 19-9 levels

We also compared the performance of the Epi-TOP Pancreatic assay with conventional serum tumor markers, as serum CA 19-9 and CEA are widely used blood markers for PDAC. Our novel Epi-TOP Pancreatic assay was more accurate than the CA19-9 and CEA assays (93.3%, 90.0%, and 90.0%, respectively). Also, the Epi-TOP Pancreatic assay showed sensitivity and specificity comparable to CA19-9 and CEA (**Table 2**; **Supplementary Table S5**). Additionally, we assessed the concordance between the methylation assay and the traditional blood tumor markers CA 19-9 and CEA. The positive and negative concordance rates between the Epi-TOP Pancreatic assay and the tumor markers CA 19-9 and CEA were determined to be 72.73%, 77.78%, and 89.47%, 85.71%, respectively. Notably, the overall concordance rates between the Epi-TOP Pancreatic assay and both blood markers reached 83.33%, as outlined in **Supplementary Table S6**. The AUC value for the combination of the 7-gene epigenetic methylation panels, *KRAS* mutation, CEA, and CA19-9 was 1.000, with a sensitivity of 95.0% and a specificity of 96.67% (**Figure 3D**).

## 4 Discussion

We investigated the diagnostic performance of a novel DNA methylation assay using Epi-sPNA technology without bisulfite for PDAC in surgically resected PDAC and normal tissues. We identified specific seven hypermethylated genes, namely *HOXA9*, *TWIST*, *WT1*, *RPRM*, *BMP3*, *NPTX2*, and *BNC1*, and developed the Epi-TOP pancreatic assay. Our panel showed high sensitivity, specificity, and accuracy. Sequentially, we performed the 7-gene panels with *KRAS* mutation via plasma cfDNA, comparing PDAC, benign pancreatic cystic neoplasm, and healthy controls. Our 7-gene epigenetic methylation panels, combined with *KRAS* mutation validation test, also showed higher sensitivity, specificity, and accuracy compared to traditional serum markers, CA19-9, and CEA. These results indicate that the Epi-TOP Pancreatic assay, along with *KRAS* mutations, holds potential as biomarkers for detecting PDAC from blood.

Diagnostic methods for PDAC have previously been studied (10, 16, 23, 24). Epigenetic alterations, which play an important role in the progression and metastasis of PDAC, manifest early in its development (7). Consequently, DNA methylation has been proposed as a promising target for identifying biomarkers for PDAC. Several methylation-based analyses using various samples, including resected tissues, pancreatic juice, and blood, have been conducted in recent years (25). Moreover, epigenetic alterations in circulating cfDNA samples have been explored as potential blood biomarkers for the diagnosis of PDAC.

Ying et al. (26) identified the *BNC1*, *ADAMTS1*, *LRFN5*, and *PXDN* genes as novel liquid biopsy-based methylation biomarkers. They developed a 4-gene methylation panel, validating it using blood cfDNA samples across all stages of PDAC and healthy controls. Similarly, Wu et al. (11) investigated PDAC, normal tissues, and plasma, creating, validating, and testing a 56-marker PDACatch assay. Both studies highlighted the high sensitivity and specificity of liquid biopsy-based methylation panels in distinguishing PDAC patients at all stages from healthy controls. However, their primary focus was on detecting PDAC in healthy controls, without differentiating PDAC and benign pancreatic disease using plasma samples. In contrast, our biomarker panel underwent sequential validation, exhibiting superior sensitivity, specificity, and accuracy in plasma cfDNA. This indicates its potential as a blood marker for PDAC, especially in distinguishing it from benign pancreatic cystic neoplasm, which can be confused with PDAC in imaging diagnostics, as well as from healthy controls.

Information on the impact of combining epigenetic DNA methylation markers with the identification of genetic *KRAS* mutations in plasma cfDNA is limited. Shinjo et al. (17) identified five DNA methylation markers, namely *ADAMTS2*, *HOXA1*, *PCDH10*, *SEMA5A*, and *SPSB4* genes, using fine-needle aspiration samples from PDAC patients. They validated a combination of these markers and *KRAS* mutations in cfDNA, reporting a low detection rate (49%) of at least one of the five markers in plasma. The combination showed improvement but relatively low sensitivity and specificity levels (68% and 86%, respectively). Apart from this study, no additional research has investigated the combination of epigenetic methylation biomarkers with genetic *KRAS* somatic mutations, which are the most common mutations in PDAC. In our study, combining a 7-gene epigenetic DNA methylation marker with *KRAS* mutation validation achieved notably high sensitivity, specificity, and accuracy (90.0%, 95.0%, and 93.3%, respectively). These results showed higher or at least equal sensitivity and specificity compared to CA19-9 and CEA. This suggests that integrating genetic and epigenetic biomarkers may provide valuable insights into the carcinogenesis of PDAC, offering a promising approach for its diagnosis.

Over the past decade, the United States Food and Drug Administration (US FDA) has granted approval for the utilization of DNA methylation biomarker assays in both colorectal and bladder cancer. The FDA-approved methylation test, Cologuard (Exact Sciences), exhibited sensitivities of 92.3% and 42.4% for patients diagnosed with colorectal cancer and advanced adenoma, respectively, and demonstrated a specificity of 86.6%, comparing the results of the commercially available fecal immunochemical test (27). Furthermore, the Bladder EpiCheck Kit (Nucleic, Ltd) showed sensitivities and specificities of 94.3% and 79.6%, respectively, for detecting bladder cancer (28). Both aforementioned tests utilize DNA isolated from stool or urine specimens, which contain biomaterials directly derived from the cancer site. However, higher heterogeneity is observed in non-blood-based samples compared with blood samples and the concentration of DNA in stool or urine is likely to be influenced by other conditions. In contrast, our assay employs blood-derived cfDNA, which may be contaminated with an excess amount of DNA from non-cancerous tissues, but could benefits sample collection and for general use of clinical samples. Taking these factors into account, we believe that our blood-based assay demonstrates utility in testing advanced-stage pancreatic cancer patients.

Traditionally, DNA methylation analysis has relied on bisulfite treatment, followed by subsequent molecular analysis techniques such as pyrosequencing and methyl-specific PCR (29–31). However, bisulfite-based methods often exhibit suboptimal diagnostic accuracy and reproducibility due to DNA template degradation during the treatment process. Additionally, these approaches typically require a substantial amount of DNA for a single analysis, rendering them impractical for liquid biopsy-derived DNA analysis, particularly for blood-based disease screening (32). To overcome this, we developed a novel technology: the Epi-TOP methylation detection method. The Epi-TOP methylation detection method employs a modified PNA probe with a higher binding affinity for methylated cytosine bases compared to non-methylated cytosine. This unique characteristic enables discrimination at a single-base resolution without necessitating prior conversion treatments like bisulfite. The methylation status or level of the target DNA molecule can be assessed using the difference in Ct values between the target gene and a control gene unaffected by methylation. This innovative approach facilitates the analysis and screening of cancer-derived cfDNA methylation using ultra-low amounts of DNA.

Our study possesses several limitations. Firstly, it was based on relatively small sample sizes, which could lead to insufficient statistical power, a lack of comparison study with traditional bisulfite-based methylation detection method, and the necessity for further validation of the assay through the incorporation of expanded sample cohorts. Secondly, it is need to validate the diagnostic role of this assay using preoperative plasma samples from patients with resectable early stage PDAC. We first investigated the diagnostic performance of the Epi-TOP Pancreatic assay in patients with stage IV PDAC because cfDNA levels are higher with greater tumor burden. This is necessary because tumor biomarkers have different roles in screening for early diagnosis, recurrence monitoring, prognostic, and predictive purposes. Therefore, further validation of this assay is warranted to investigate its diagnostic performance in patients with early-stage PDAC. Thirdly, there is a growing trend in whole genome-wide DNA methylation sequencing models for PDAC diagnosis. While a genome-wide DNA methylation analysis might provide broader insights, our study’s focus was not on screening novel targets but on selecting combination of the hypermethylated differentially methylated regions (DMRs) associated with PDAC which showing highest detection accuracy and have been extensively reported that having diagnostic ability. To accomplish this, we employed a RT-PCR assay capable of analyzing methylation levels of over 60 tumor suppressor gene promoters previously reported to be implicated in cancer development. Our aim was to identify PDAC-specific hypermethylated DMRs from these well-established genes rather than discovering novel epigenetic markers.

Our study suggests that a combination of seven differentially methylated genes, along with *KRAS* mutations, may serve as potential biomarkers for diagnosing PDAC through blood-based liquid biopsy. This innovative assay has the potential to serve as a diagnostic biomarker for PDAC, potentially improving outcomes and the quality of life for individuals diagnosed with PDAC.

## Data availability statement

The raw data supporting the conclusions of this article will be made available by the authors, without undue reservation.

## Ethics statement

The studies involving humans were approved by The Institutional Review Board (IRB No. 2023-06-010) at Chungbuk National University Hospital. The studies were conducted in accordance with the local legislation and institutional requirements. The participants provided their written informed consent to participate in this study.

## Author contributions

HK: Conceptualization, Data curation, Formal analysis, Funding acquisition, Investigation, Methodology, Project administration, Resources, Software, Supervision, Validation, Visualization, Writing – original draft, Writing – review & editing. JC: Data curation, Methodology, Writing – review & editing. I-GD: Data curation, Methodology, Writing – review & editing. Y-PL: Data curation, Writing – review & editing. HKK: Data curation, Writing – review & editing. YY: Data curation, Writing – review & editing. JK: Data curation, Writing – review & editing. KL: Data curation, Writing – review & editing. CB: Formal analysis, Methodology, Software, Supervision, Validation, Visualization, Writing – review & editing. EJ: Formal analysis, Methodology, Software, Supervision, Validation, Visualization, Writing – review & editing. KK: Formal analysis, Methodology, Software, Supervision, Validation, Visualization, Writing – review & editing. HH: Conceptualization, Data curation, Formal analysis, Funding acquisition, Investigation, Methodology, Project administration, Resources, Software, Supervision, Validation, Visualization, Writing – original draft, Writing – review & editing.

## Supplementary material

Supplementary Table 1 Summary of methylation status of the seven genes.

Supplementary Table 2 Diagnostic performance of the Epi-TOP pancreatic assay in the discovery tissue set.

Supplementary Table 3 Methylation status based on clinicopathological features in the discovery tissue set.

Supplementary Table 4 Diagnostic performance of the Epi-TOP pancreatic assay with *KRAS* mutation in the validation plasma set.

Supplementary Table 5 Comparison of Epi-TOP Pancreatic assay with *KRAS* mutation, CEA and CA19-9 in the validation plasma set.

Supplementary Table 6 Correlation rates between the results of Epi-TOP Pancreatic assay with *KRAS* mutation and traditional blood-based tumor markers in the validation plasma set.
