# Supplementary material for: Novel diagnostic biomarkers for pancreatic cancer: assessing methylation status with epigenetic-specific peptide nucleic acid and KRAS mutation in cell-free DNA
Source: Front Oncol. 2024 Jul 5;14:1395473. doi: 10.3389/fonc.2024.1395473 (PMC11257850; doi:10.3389/fonc.2024.1395473)
Supplement: Supplementary Table 1 — Summary of methylation status of the seven genes. [file Table_1.docx]

**Table S1.** Summary of methylation status of the seven genes

| Gene | Discovery set  (Tissue) | |  | Validation set  (Plasma cfDNA) | | |
| --- | --- | --- | --- | --- | --- | --- |
|  | PDAC | Normal pancreas |  | PDAC | Benign pancreatic cystic neoplasm | Healthy |
| *HOXA9* | 34.8% | 6.5% |  | 70.0% | 20.0% | 0% |
| *TWIST* | 47.8% | 13.0% |  | 20.0% | 0% | 0% |
| *WT1* | 63.0% | 2.2% |  | 40.0% | 30.0% | 10.0% |
| *RPRM* | 32.6% | 2.2% |  | 10.0% | 10.0% | 0% |
| *BMP3* | 78.3% | 6.5% |  | 30.0% | 0% | 0% |
| *NPTX2* | 71.7% | 2.2% |  | 90.0% | 20.0% | 0% |
| *BNC1* | 65.2% | 0% |  | 40.0% | 0% | 0% |

PDAC, pancreatic ductal adenocarcinoma; cfDNA, cell-free DNA

**Table S2.** Diagnostic performance of the Epi-TOP pancreatic assay in the discovery tissue set

|  |  | PDAC | Normal pancreas | Total |
| --- | --- | --- | --- | --- |
| Epi-TOP pancreatic assay | Positive | 43 | 2 | 45 |
|  | Negative | 3 | 44 | 47 |
|  | Total | 46 | 46 |  |
| Sensitivity (%) (95% CI) | | 93.48 (82.10–98.63) | | |
| Specificity (%) (95% CI) | | 95.65 (85.16–99.47) | | |
| Accuracy (%) (95% CI) | | 94.57 (87.77–98.21) | | |

PDAC, pancreatic ductal adenocarcinoma; CI, confidence interval

**Table S3.** Methylation status based on clinicopathological features in the discovery tissue set

| Clinicopathological features (No. of patients) | | Total number of methylated markers | Average number of methylated markers per person |
| --- | --- | --- | --- |
| Gender |  | |  |
| Male (24) | 91 | | **3.79** |
| Female (22) | 90 | | **4.09** |
| Age, years |  | |  |
| <60 (12) | 50 | | **4.17** |
| 60≤70 (13) | 49 | | **3.77** |
| 71≤ (21) | 82 | | **3.90** |
| Stage |  | |  |
| IB (3) | 11 | | **3.67** |
| IIA (6) | 25 | | **4.17** |
| IIB (28) | 117 | | **4.18** |
| III (9) | 28 | | **3.11** |

**Table S4.** Diagnostic performance of the Epi-TOP pancreatic assay with *KRAS* mutation in the validation plasma set

|  |  | PDAC | Benign cystic neoplasm and healthy | Total |
| --- | --- | --- | --- | --- |
| Epi-TOP pancreatic assay with *KRAS* mutation | Positive | 9 | 1 | 10 |
|  | Negative | 1 | 19 | 20 |
|  | Total | 10 | 20 | 30 |
| Sensitivity (%) (95% CI) | | 90.00 (55.50–99.75) | | |
| Specificity (%) (95% CI) | | 95.00 (75.13–99.87) | | |
| Accuracy (%) (95% CI) | | 93.33 (77.93–99.18) | | |

PDAC, pancreatic ductal adenocarcinoma; CI, confidence interval

|  | Epi-TOP Pancreatic assay with *KRAS* mutation | CA19-9 | CEA |
| --- | --- | --- | --- |
| Sensitivity (%) (95% CI) | 90.00 (55.50–99.75) | 90.00 (55.50–99.75) | 80.00 (44.39–97.48) |
| Specificity (%) (95% CI) | 95.00 (75.13–99.87) | 90.00 (68.30–98.77) | 95.00 (75.13–99.87) |
| Accuracy (%) (95% CI) | 93.33 (77.93–99.18) | 90.00 (73.47–97.89) | 90.00 (73.47–97.89) |

**Table S5.** Comparison of Epi-TOP Pancreatic assay with *KRAS* mutation, CEA and CA19-9 in the validation plasma set

CA19-9, Carbohydrate antigen 19-9; CEA, Carcinoma embryonic antigen; CI, confidence interval

**Table S6**. Correlation rates between the results of Epi-TOP Pancreatic assay with *KRAS* mutation and traditional blood-based tumor markers in the validation plasma set

|  | Epi-TOP Pancreatic assay with  *KRAS* mutation and CA19-9 | Epi-TOP Pancreatic assay with *KRAS* mutation and CEA |
| --- | --- | --- |
| Positive concordance rate (%) (95% CI) | 72.73 (39.03-93.98) | 77.78 (39.99-97-19) |
| Negative concordance rate (%) (95% CI) | 89.47 (66.86-98.70) | 85.71 (63.66-96.95) |
| Overall concordance rate (%) (95% CI) | 83.33 (65.28-94.36) | 83.33 (65.28-94.36) |

CA19-9, Carbohydrate antigen 19-9; CEA, Carcinoma embryonic antigen; CI, confidence interval
